# Round the Hospitals

**Published:** 1916-12-23

**Authors:** 


### ROUND THE HOSPITALS.

The founding of the College of Nursing is awakening interest in many features of nurse training which have heretofore been ignored or ill appreciated. The College being now accepted as a certainty, attention is being directed to those branches of the profession most affected by trained nurses on obtaining their certificate. With a view to encourage inquiry in England on this point, we have pleasure in giving the occupations of the last seventy-five nurses, who have graduated from the Massachusetts General Hospital, Boston. Six are at home, seven are private nurses, four are instructors, four specialising in surgery, three in social service work, four in public health nursing, twenty-four are head nurses of wards or departments, one is an anæsthetist, one a superintendent of a hospital, one a superintendent of a training-school, and twenty are assistant superintendents of hospitals or training-schools. It is fair to conclude from this return that the average standard of education and the quality of the graduates of the Massachusetts General Hospital is distinctly high. In England at the larger hospitals with training-schools from eighty to 150 nurses graduate each year. It would be interesting if the superintendents, or some of them, would kindly send us the actual occupations and positions held by the nurse-graduates from their school during the last completed year. If these returns could be made fairly complete they could not fail to be a help to the Council of the College.

We were struck by the thoroughness which characterises the training of nurses in the best American hospitals. We had the privilege of attending the class at one general hospital where the superintendent was lecturing on chemistry. Her system was to speak with great distinctness and clearness and to impress upon each student the importance of taking full notes of the lectures. This struck us as being a very direct test of the average education and ability of the members of the class. The accuracy of this conclusion was proved, when, in reply to a request, several of the probationers under instruction handed up their note-books, which we examined carefully and with great interest. The notes were clearly written, accurately expressed, and complete records, including as they did a number of formulæ, which exhibited a power of apprehension that did the teacher and class infinite credit. There can be no question that the teaching of cookery and dietetics in the United States is far in advance of that in the majority of schools in the United Kingdom. There is an admirable system, however, where remarkably efficient class work is done, at the Western Infirmary, Glasgow. Both are so thorough, and so admirably organised as to encourage those interested to make a point of going to Glasgow and master both thoroughly.

The establishment of the College of Nursing makes it imperative that the system of teaching and organisation of the classes, covering the many subjects now embraced in the hospital curriculum for nurses, shall attain a high standard of excellence at every British nurse-training school. Then too examinations may take a very important position in the years of training, so far as the nurse-pupils are concerned. There is a general inclination to adopt a one-portal examination. This can be carried out fully in regard to theoretical examinations, but to it will have to be added a plan, providing for thorough examination in regard to practical points, which it may be found difficult, if not impossible, to include within a one-portal examination. The best solution will probably be to adopt the system at the London University and lay down as a basis for the examination of nurse-pupils a system of internal and external examinations. Everyone interested in the College and the training of nurses has here a subject well worthy of their consideration. We hope that amongst our readers will be found some superintendents and teachers who have experience and ideas on these subjects, which they will formulate and send to us for publication in due course.

The truth is that there is a marked difference between those responsible for the training and education of nurses in the American schools and those in Great Britain. In the former the heads are mostly women of action who take the greatest interest in the whole question of nurse-training, education, and examination, who have clear ideas on the subject and who are willing to set them down and supply them for the benefit of the nursing profession, either for publication in one of the technical journals, or for the information and guidance of those of their sisters who are new to the work, and who wish to have the benefit of the matured experience of the responsible teacher in one of the principal schools. No doubt Teachers' College has helped our sisters materially, under Miss Nutting's experienced direction, in the nursing field of the United States. We hope that the birth of the College of Nursing may have the same good results in Great Britain. One step towards these results might be that several of the superintendents or members of their staff in the greater nurse-training schools of this country should send us their views on these important matters. It would be interesting, in this connection, if some of these leaders in British nursing would formulate their opinion and experience of (*a*) how to train for the work of an examiner, and (*b*) how to keep examiners in touch with current developments.

The Poor-Law nurses of Birmingham and district have unanimously decided, through their representatives, not to form a nursing section of the National Poor-Law Officers' Association. This decision is important in view of the strenuous efforts which are being made to recruit nurses for this Association by means of affiliated nurses' sections in Poor-Law districts. In our issue of September 9 (p. 547) we dealt fully with the reasons why, in our opinion, this Association cannot represent trained Poor-Law nurses. Is there, indeed, any present necessity for a special organisation of Poor-Law nurses with a view to the protection of their class interests? Will some of them write us their views on this point?

